# Lockwood’s Practice of Medicine

**Published:** 1902-02

**Authors:** 


LOCKWOOD'S PRACTICE OF MEDICINE.—A MANUAL OF THE PRACTICE OF MEDICINE.—By George Roe Lockwood, M. D., Professor of Practice in the Woman's Medical College of the New York Infirmary. Second edition, revised and enlarged. Octavo volume of 847 pages, with 79 illustrations and 20 full-page plates. Philadelphia and London: W. B. Saunders & Company. 1901. Cloth, $4.00, net.

The author has adopted in this manual a method of classification similar to that used by Osler, which represents a decided improvement upon the older methods of arrangement.

The work contains in a compact and readable form all of the essential facts of the etiology, pathology, symptomatology, prognosis and treatment usually found in text-books on practice. It is a valuable book to have at hand to be used for quick reference in connection with one of the large works.

The mechanical part of the work is deserving of the highest praise. R.

